# Logicality and the picture theory of language

**DOI:** 10.1007/s11229-024-04549-4

**Published:** 2024-04-16

**Authors:** Tue Trinh

**Affiliations:** 1 Leibniz-Zentrum Allgemeine Sprachwissenschaft, Pariser Straße 1, Berlin, 10719 Germany; 2 Institut für Philosophie, Humboldt-Universität zu Berlin, Unter den Linden 6, 10099 Berlin, Germany

**Keywords:** Logicality, Grammar, Trivialities, Wittgenstein, Tractatus

## Abstract

I argue that there is tension in Wittgenstein’s position on trivialities (i.e. tautologies and contradictions) in the Tractatus, as it contains the following claims: (A) sentences are pictures; (B) trivialties are not pictures; (C) trivialities are sentences. A and B follow from the “picture theory” of language which Wittgenstein proposes, while C contradicts it. I discuss a way to resolve this tension in light of Logicality, a hypothesis recently developed in linguistic research. Logicality states that trivialities are excluded by the grammar, and that under the right analysis sentences which look trivial are in fact contingent. The tools necessary to support Logicality, I submit, were not available to Wittgenstein at the time, which explains his commitment to C. I end the paper by commenting on some points of contact between analytic philosophy and the generative enterprise in linguistics which are brought into relief by the discussion.

## The picture theory of language

Wittgenstein (1921), commonly known as Tractatus Logico-Philosophicus,^1^ purports to have found the conclusive solution to all philosophical problems (“die Probleme endgültig gelöst zu haben”). This solution consists in showing that there are really no problems, or more precisely, that the formulation of these problems (“die Fragestellung dieser Probleme”) will be recognized as gibberish, once the logic of our language (“die Logik unserer Sprache”) is understood.

The main contribution of the Tractatus, in my opinion, is the thesis that “the logic of our language” is to be identified with what has traditionally been called “grammar”. Grammar, in the classic sense, is what distinguishes between sentences and non-sentences.^2^ It defines what a sentence is, thereby delineating what can be expressed as either true or false. This is precisely Wittgenstein’s stated objective in the Tractatus. “The book [...] aims to draw a limit [...] to the expression of thoughts,” he says in the preface. This limit, as he subsequently clarifies, will be drawn “in language”, and will serve to separate the intelligible from “nonsense”.^3^

But what does identifying logic with grammar amount to exactly? To repeat: grammar distinguishes between sentences and non-sentences. It accounts for the fact that (1a) is a sentence but (1b) is not, for example. 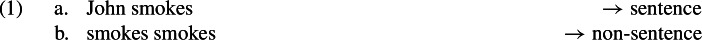


Logic, on the other hand, is what distinguishes between valid and invalid arguments. It accounts for the fact, for example, that the inference in (2a) is justified but the inference in (2b) is not. 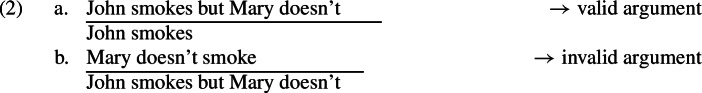


The suprising claim made by Wittgenstein in the Tractatus is that the phenomenon exemplified by (1) and the phenomenon exemplified by (2) should receive a unified explanation. Specifically, the contrast with respect to sentencehood and the contrast with respect to validity should be accounted for by one and the same theory. This theory will tell us what a sentence is and, in addition, will tell us, for any sentence $\phi$, what other sentences are true if $\phi$ is true. Last but certainly not least, it will also dissolve all philosophical problems.

The theory in question is known as the “picture theory of language” (Keyt, 1964), henceforth PTL. The essence of PTL is presented in 4.01: “A sentence is a picture of reality”.^4^ Sentences, Wittgenstein maintains, are pictorial. They represent states of affairs in the same way as, say, the score of Schubert’s Unvollendete represents the sound of this symphony: “A gramophone record, the musical thought, the musical notation, the sound waves, all stand to one another in that internal relation of depicting that holds between language and world” (4.014). The structure of a sentence, then, is isomorphic to the structure of the state of affairs it describes (Daitz, 1953): “The configuration of simple signs in a propositional sign corresponds to the configuration of objects in a state of affairs” (3.21). To see how PTL accounts for both sentencehood and validity, we will discuss an example.^5^

Suppose the linguistically relevant reality has three basic elements, i.e. three “objects” (“Gegenstände”): (i) John, (ii) Mary, and (iii) the property of smoking.^6^ Logical space would then contain four possible worlds: one where both John and Mary smoke, one where only John smokes, one where only Mary smokes, and one where neither of them smokes. Let us now consider two languages. The first is familiar: John is symbolized as *j*, Mary as *m*, smoking as *s*, that John smokes as *s*(*j*), that Mary doesn’t smoke as $\lnot s(m)$, that John smokes but Mary doesn’t as $s(j) \wedge \lnot s(m)$, etc. We will call this language L_F_, with the subscript “F” being mnemonic for ‘Fregean’. The second language, call it L_W_, will be the one which approaches more closely the “pictorial” ideal envisioned by Wittgenstein. In L_W_, John is symbolized as a pebble , Mary as a marble , and smoking as a jar . The fact that an individual smokes or does not smoke will be represented by placing the symbol for that individual inside or outside of the symbol for smoking, respectively. We now compare some sentences in the two languages as to the correspondence between them and the states of affairs they describe.^7^
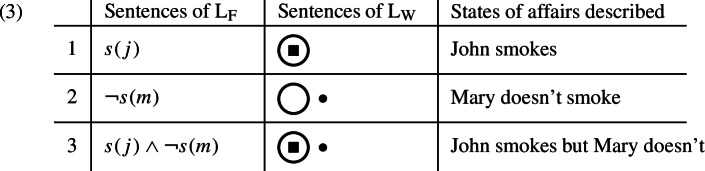


Let us ask in what sense L_W_ is “pictorial”, i.e. “isomorphic” to reality, but L_F_ is not. First, note that a necessary condition for the isomorphism between two structures is that they have the same number of primitive elements. This condition is satisfied by L_W_ but not L_F_. The reality to be represented has three objects: John, Mary, and smoking. L_W_ has three basic symbols, i.e. names: the pebble, the marble, and the jar. This is not the case for L_F_, which has seven names: *j*, *m*, *s*, (, ), $\lnot$, and $\wedge$. Sentences of L_W_ contain nothing but names hanging in one another “like the links of a chain” (2.03), i.e. by virtue of their form alone, just as in the represented states of affairs objects hang in one another without any kind of “logical frame” into which they fit (Hintikka, 1994). Sentences of L_F_, on the other hand, contain not only names but also “syncategorematic glue” (i.e. the brackets and the connectives) which holds them together. There is another sense in which L_W_ is more pictorial than L_F_. Consider the shape of the names. In L_F_, the name for smoking is *s*, a letter, just like the names for John (*j*) and Mary (*m*). In L_W_, it is the jar, which differs from the pebble and the marble in that it is a container which allows the distinction to be made between things that are inside and things that are outside of it. The property of smoking, by nature, distinguishes between smokers and non-smokers. There is thus a higher degree of verisimilitude, in the relevant sense, between smoking and the jar than between smoking and the letter *s*.^8^

This last point about the shapes of names brings us to the issue of sentencehood. The claim, to repeat, is that PTL accounts for sentencehood: it includes sentences and excludes non-sentences. Let us start by considering what would be non-sentences in L_F_: *j*(*s*), *s*(*s*), *m*(*j*), etc. How are these excluded? Obviously, the shape of the symbols themselves would not do the job: writing *s*(*j*) is just as easy as writing *j*(*s*). What would be required is an extrinsic theory of syntax which (i) categorizes the basic symbols into different “parts of speech”, i.e. “types”, and then (ii) imposes rules of combination upon these types. For example, it would say that *s* is an adjective, *j* is a noun, and if $\alpha$ is an adjective and $\beta$ is a noun then $\alpha (\beta )$ is a sentence but $\beta (\alpha )$ is not a sentence, etc.^9^ Let us now turn to L_W_. What would be the L_W_ counterpart of the non-sentence *j*(*s*)? Well, it would be the jar placed inside the pebble. But we cannot place the jar inside the pebble: the forms of these symbols do not allow such a configuration. Thus, the pictorial counterpart of *j*(*s*) is ineffable.^10^ The same holds for the pictorial counterparts of *s*(*s*) and *m*(*j*): there is no way to place the jar inside of itself or the pebble inside of the marble. As we can see, there is no need for an extrinsic theory of syntax. The shapes of the symbols themselves determine which combinations are possible and which ones are not, thereby distinguishing between sentences and non-sentences. Grammar, thus, emerges from the pictorial nature of representation: PTL accounts for sentencehood.

How does PTL account for validity? Again, we first consider L_F_. What guarantees, for instance, that L_F_-3 entails L_F_-1 but not vice versa? Obviously, it cannot be the shape of the expressions themselves. The logic of L_F_, just like its syntax, has to be imposed extrinsically by way of a theory which tells us that the inference in (4a) is valid but the inference in (4b) is not.^11^
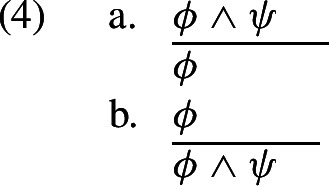


Let us now ask a parallel question for L_W_: what guarantees that L_W_-3 entails L_W_-1 but not vice versa? The answer seems to be that the question itself is ridiculous. I can see, with my own eyes, that the pebble is inside of the jar in L_W_-3. There is no way for me not to see the pebble inside the jar when I see the pebble inside and the marble outside the jar. In other words, I cannot conceive of the possibility that L_W_-3 is true while L_W_-1 is false, simply because I see L_W_-1 in L_W_-3. What about the fact that L_W_-1 does not entail L_W_-3? Well, this fact is also obvious: looking at L_W_-1, I do not see the marble, which means I do not see the marble outside the jar, which means I do not see both the pebble inside the jar and the marble outside the jar. In other words, I do not see L_W_-3 in L_W_-1.^12^ If we had to posit “rules” which allow the inference from L_W_-3 to L_W_-1 but disallow the inference in the opposite direction, they would say something banal and empty like “Look!” or “Think!” Thus, rules of inference are “superfluous” for L_W_ (5.132). “That the truth of one proposition follows from the truth of other propositions can be seen from the structure of the propositions” (5.13). Therefore, logic “take[s] care of itself” (5.473) (cf. McGinn, 2006). Logic, as it were, emerges from the pictorial nature of representation: PTL accounts for validity.

Finally, how does PTL dissolve all philosophical problems? Here is Wittgenstein’s reasoning, as I see it. First, a “problem” exists only to the extent that it can be formulated. How is a problem formulated? By asking whether a sentence is true, which means, given PTL, asking whether a picture corresponds to reality. But pictures are empirical: we can only know whether they are true by observation (2.224, 2.225). This means that every problem can in principle be settled by observation, which is to say by natural science. Philosophy, however, “is not one of the natural sciences” (4.111). It then follows that there are no problems for philosophy to solve.

My main aim in the preceding paragraphs has been to argue that Wittgenstein’s picture theory of language (PTL) serves the double function of (i) distinguishing between sentences and non-sentences and (ii) distinguishing between valid and invalid arguments. I will now turn to a discussion on sentences that are either a contradiction or a tautology. Following a practice common in the linguistic literature, I will call these sentences “trivialities”. This, I should note, is not a term Wittgenstein uses in the Tractatus. My use of it is purely to reduce the number of words on the page: instead of saying “contradictions and tautologies” every time, I say “trivialities”.

Let us start, again, with L_F_. Take $s(j) \wedge \lnot s(j)$, for example. This is a contradiction. It is, of course, a sentence of L_F_, given that the syntax of L_F_ should (i) include *s*(*j*) as a sentence, (ii) allow any sentence to be negated, and (iii) allow any two sentences to be conjoined.^13^ It follows, then, that the negation of $s(j) \wedge \lnot s(j)$, i.e. $\lnot (s(j) \wedge \lnot s(j))$, a tautology, is also a sentence.^14^ Thus, trivialities are sentences of L_F_. Now let us ask what the L_W_ counterpart of $s(j) \wedge \lnot s(j)$ would be. Presumably, it would be a configuration in which the pebble is inside as well as outside of the jar. This configuration, of course, is ineffable. Thus, contradictions in L_F_ have no “translation” in L_W_. The shapes of the symbols in L_W_ just do not allow such arrangements. What about tautologies, which are the negation of contradictions? Do they have L_W_ translations? I propose that the answer is no. Note that tautologies are the negation of contradictions. Let us make the conjecture in (5). 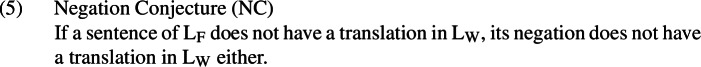


I will now make a brief excursus on NC. First, I emphasize that it is a conjecture. At this point, I do not have a general account of how negation works for such a pictorial language as L_W_. The cases we have considered are simple: $\lnot S(m)$ is represented by placing the marble outside of the jar, for example. But how do we represent $\lnot (S(j) \wedge \lnot S(m))$? I do not have an answer. It is intuitively clear how conjunction is represented pictorially. But that is not the case for negation. And it is not clear to me, from what Wittgenstein says in the Tractatus, whether he has a cogent and general proposal with respect to pictorial negation. Hintikka (2000) and Eisenthal (2023) sketch some ideas which are, in my opinion, quite vague, and to the extent that I can make out what these authors say, I do not see how they would represent $\lnot (S(j) \wedge \lnot S(m))$. Note that once we figure out how negation works pictorially, we will also have disjunction and implication, as these functions are definable in terms of negation and conjunction. For present purposes, I will assume NC, and leave the task of deriving this conjecture, either from Wittgenstein’s text or from elaborations of it, to future work. My hope is that the readers will not find NC so implausible as to stop reading.^15^ This is the end of the excursus.

Let us now come back to the main discussion. Given NC and the fact that contradictions are ineffable in L_W_, it follows that tautologies are also ineffable in L_W_. Thus, L_W_ excludes contradictions as well as tautologies, i.e. it excludes trivialities, from the set of sentences. This fact, of course, lies in the nature of pictures. A picture, as was mentioned above, is empirical: it has to match some state of affairs and fail to match some other. Thus, it cannot be trivial. Just as we cannot use musical notation to produce a score which matches every or no possible piece of music, we cannot use linguistic signs to construct a model of every or no possible state of affairs. At various places in the Tractatus, Wittgenstein seems to concede that trivialities are non-sentences. “Tautology and contradiction are not pictures of reality”, he admits in 4.462. It should then follow that they are non-sentences, since sentences, by hypothesis, are pictures of reality. “[P]ropositions that are true for every state of affairs cannot be combinations of signs at all”, Wittgenstein says in 4.466. And because they are not combinations of signs, their negations are not either: “Tautology and contradiction are the limiting cases of the combination of symbols, namely, their dissolution” (4.466). But Wittgenstein, curiously, stops short of considering trivialities non-sentences. He calls non-sentences, i.e. arrangement of signs that are excluded by logical syntax, “nonsensical” (“unsinnig”). For example, *John likes* and *Socrates is identical* would be nonsensical, because in both cases a transitive predicate is lacking its direct object, incurring a violation of what linguists call the $\Theta$-Criterion.^16^ However, Wittgenstein insists that that is not the case with trivialities. He declares, explicitly, that “tautology and contradiction are [...] not nonsensical”, and confirms that they are “part of the symbolism” (4.4611). Thus, he takes trivialities to be sentences. This is baffling: given the theory Wittgenstein proposes (PTL), he should exclude trivialities from the symbolism, i.e. should consider them nonsensical, but he does not. It should be noted, nevertheless, that Wittgenstein does end up describing trivialities using a word which is very close to “nonsensical”. The word is “senseless” (“sinnlos”). “Tautology and contradiction are senseless”, he says in 4.461. Thus, non-sensicality and senselessness both implies non-interpretability, but only non-sensicality implies non-sentencehood (von Wright, 2006; Biletzki & Matar, 2021; Proops, to appear). The morphological and semantic similarity between “senseless” and “non-sensical”, I think, is not accidental. It suggests that Wittgenstein finds trivialities to be “almost” non-sentences. One might say he thinks they should be non-sentences but refrain from going as far as saying they actually are.

The question that arises at this point is whether there is inconsistency in Wittgenstein’s theory of language. I will consider, first, the negative answer. Suppose we say there is no inconsistency. The challenge we face would then be to make sense of the fact that Wittgenstein says 
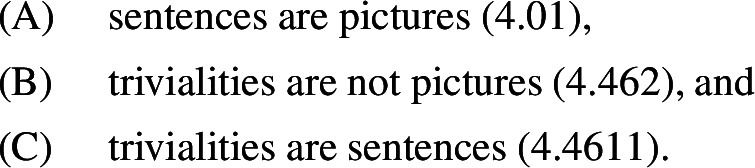


How would we do that? Well, let us claim that A has existential, not universal, quantificational force. Specifically, the word “sentences” in A means ‘some sentences’, not ‘all sentences’.^17^ Under this reading, A is compatible with B and C. This account, of course, requires us to say that PTL does not apply generally to all sentences. We need to exclude trivialities from its domain. One way to do that is to say that PTL applies only to what Wittgenstein calls “sinnvolle Sätze” (meaningful sentences). Trivialities are “sinnlos”, hence are not in the domain of PTL. Another way to exclude trivialities from PTL is to restrict PTL to atomic sentences. Trivialities cannot be atomic, since they involve at least one sentence and its negation, so they will not be in the domain of PTL. Restricting the domain of PTL, therefore, is a strategy to save Wittgenstein from inconsistency. It is not my aim, in this paper, to provide a conclusive argument for or against this strategy. I would just note here that I do not think it squares well with the text. Consider Wittgenstein’s use of the phrase “sinnvoller Satz”, for example. It is quite clear from the text that the adjective “sinnvoll” is not pleonastic: it restricts the set of sentences to a proper subset.^18^ But this fact strongly suggests that Wittgenstein’s use of “Satz” is not restricted. In other words, it suggests that the null hypothesis is correct: when Wittgenstein says “Satz”, he means ‘Satz’, and when he says “sinnvoller Satz”, he means ‘sinnvoller Satz’. Consequently, when he says “[d]er Satz is ein Bild der Wirklichkeit” (“sentences are pictures of realities”), he is not talking about a specific subset of sentences, but about sentences tout court.^19^ In fact, Eisenthal (2023) has argued, quite convincingly in my opinion, that there is textual evidence from both the Tractatus and Wittgenstein’s Notebooks (Wittgenstein, 1989) that “Wittgenstein consistently treated the picture theory as applying to all propositions” (Eisenthal, 2023, p. 165), and that “in many of Wittgenstein’s canonical statements to the effect that propositions are pictures, the claim is put forward in complete generality” (Eisenthal, 2023, p. 167).

Thus, while I acknowledge, and do not rule out, the possibility of making sense of A, B and C by interpreting A as an existential statement, i.e. by restricting the domain of PTL, I would like to explore an alternative. This alternative does not ask how A can be interpreted so that Wittgenstein’s theory is consistent, but accepts that the theory is inconsistent and asks why Wittgenstein commits to C. Note, first, that A and B are not inconsistent: they both follow from PTL. It is C in conjunction with A and B that causes a problem. Second, note that I am not claiming Wittgenstein is unaware of the inconsistency. I believe he is. He proposes a theory of language which is, in a way, overwhelmingly intuitive: symbols stand for objects, and configurations of symbols stand for configurations of objects. This is A. It is also overwhelmingly intuitive that such pictorial representations cannot be a priori true or a priori false, i.e. cannot be trivial. Wittgenstein realizes this, and stresses it in the text (2.225). This is B. The consequence of A and B is clearly that trivialities is not part of the language, because they cannot be pictured. Wittgenstein, however, says they are part of the language. This is C. Is he aware that C conflicts with A and B? I think he is, and I think that is why he calls trivialities “sinnlos”. Thus, they are sentences that are “defective” in some sense. One can say: they are halfway between “sinnvoll” and “unsinnig”. But this move by Wittgenstein, in my view, does not solve the problem. Grammar specifies a set of sentences. Something is either in the set or not. Saying that trivialities are in the set but defective does not resolve the conflict between A and B on the one hand, which together entail trivialities are not in the set, and C on the other, which says they are. Thus, what I am saying is not that Wittgenstein is unaware of the inconsistency in his theory, but that he tries but in the end fails to resolve it.^20^

Let us now ask why Wittgenstein commits to C, which contradicts the theory he proposes. The reason, in my view, has a phenomenological component as well as a logical one. On the one hand, Wittgenstein experiences such expressions as (6a) and (6b) as sentences of the language, i.e. as structures which can be built using the rules of the grammar. 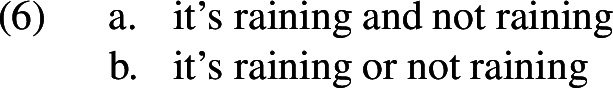


One the other hand, he interprets them as trivialities: he analyzes (6a) as $\phi \wedge \lnot \phi$, a contradiction, and (6b) as $\phi \vee \lnot \phi$, a tautology. The experience and the interpretation, together, lead to Wittgenstein’s conclusion that trivialities are sentences, which is inconsistent with the rest of his theory. Restoring consistency to Wittgenstein’s position, then, requires contesting this conclusion, which means either contesting the experience or contesting the interpretation. There is, of course, no point in the first course of action: visceral experiences such as those pertaining to sentencehood are basic facts from which we start. It is left, then, to question Wittgenstein’s analysis of (6a) and (6b). I will now turn to the discussion of a hypothesis about natural language which says that this analysis is incorrect. More specifically, it says that (6a) and (6b) are perceived as sentences precisely because they are not trivial.

## Logicality

The hypothesis in question goes by the name of “Logicality”. Logicality states that universal grammar interfaces with a natural deductive system and filters out sentences expressing tautologies or contradictions (Chierchia, 2006; Del Pinal, 2019, 2022). In other words, Logicality claims that trivialities are ill-formed. I will present this intriguing thesis by discussing two cases, one illustrating the ill-formedness of contradictions, the other that of tautologies. Both pertain to the distribution of quantifiers, exemplified by universal *every* and existential *a*.

Before I proceed, a terminological clarification is in order. In section 1, I said grammar distinguishes between “sentences” and “non-sentences”. From now on I will speak of this distinction as one between “well-formed sentences” and “ill-formed sentences”, or equivalently, between “grammatical sentences” and “ungrammatical sentences”. Thus, the notion of sentencehood will be verbalized as one of “well-formedness” or “grammaticality”. Nothing of substance hinges on this change of labels, which is made just to bring our discussion closer to how linguists talk. The fact remains that some but not all arrangements of symbols are part of the language and grammar separates those that are (“sentences”, “well-formed sentences”, “grammatical sentences”) from those that are not (“non-sentences”, “ill-formed sentences”, “ungrammatical sentences”).^21^

Let us start by considering the contrast in (7). Following standard practice, I will mark ill-formedness with #. 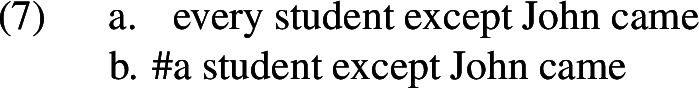


A fact about exceptive constructions, i.e. sentences of the form D(P except E)(Q), is that they are well-formed only if D, the determiner, is universal.^22^ When D is existential, ill-formedness results.

This fact suggests that interpretation can be relevant for grammaticality. I will assume the standard semantics of quantifiers according to which they express relations between two sets. For *every* the relation is ‘is a subset of’, and for *a*, ‘has a non-empty intersection with’ (Barwise & Cooper, 1981; Heim & Kratzer, 1998).^23^
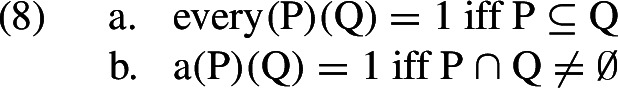


Our intuition about (7a) is that it is a normal sentence, i.e. something that could be said and understood. On the other hand, we feel that (7b) is just gibberish: it sounds weird, and we don’t really know what it means. The most well-known account of the contrast is one proposed by von Fintel (1993). What is interesting about this account, and pertinent to our discussion, is that it explains the ill-formedness of (7b) as resulting from this sentence being contradictory. Specifically, von Fintel assigns to exceptive constructions the semantics in (9), where X−Y is the complement of Y in X, i.e. the set of things in X that are not in Y. 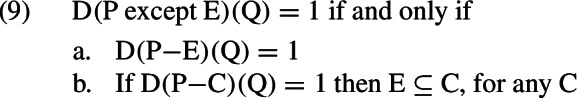


This analysis accomplishes two things at once. First, it captures the right meaning of (7a). Second, it determines (7a) to be contingent and (7b) to be trivial, thereby making a crucial distinction between the well-formed and the ill-formed sentence. Let us see how. Consider (7a) first. Given (9), this sentence is predicted to have the following truth condition.^24^
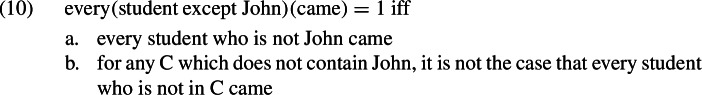


Together, (10a) and (10b) means that (i) John is a student, (ii) John did not come, and (iii) every other student came, as the reader is invited to verify for herself. This is precisely the truth condition which we intuitively associate with (7a). It is, of course, contingent: we get truth if John is the only student who did not come and falsehood otherwise. Note that C in (10b) ranges over all sets including $\emptyset$. As X$-$ $\emptyset$ $=$ X, we predict (11) to be an entailment of (10b), hence an entailment of (7a). 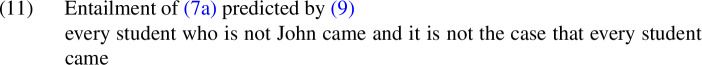


Now let us apply (9) to (7b). The truth condition we get for this sentence is (12). 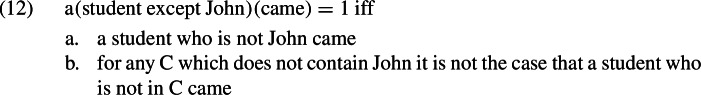


This condition, as it turns out, is trivial. Recall, again, that C can be $\emptyset$. Thus, we predict (13) to be an entailment of (12b), hence an entailment of (7b). 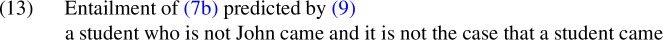


Obviously, (13) is contradictory: its first conjunct entails that a student came while its second conjunct says that this is not the case. This fact is considered by von Fintel to be responsible for the ill-formedness of (7b).^25^

Let us now turn to an example which illustrates ill-formedness of a sentence due to its being a tautology. Consider the contrast between the two existential sentences in (14).^26^
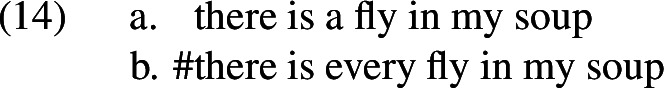


Again, we see two sentences which are identical with respect to syntactic category and constituency: they are both of the form *there*(D(P)).^27^ What distinguishes them is the semantics of D: existential in (14a), universal in (14b). This case, then, is also one where interpretation seems to affect grammaticality, as the difference between (14a) and (14b) feels like that between a well-formed and an ill-formed sentence. The standard account for (14) is Barwise and Cooper (1981). What it amounts to, basically, is that the word *there* says that its argument is true of the universe of discourse U, i.e. the set of all entities. This semantics is given in (15). The truth condition predicted for (14a) and (14b) are spelled out in (15a) and (15b), respectively.^28^
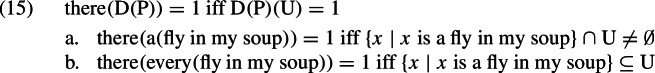


What (15a) says is that the set of flies in my soup is not empty. This condition, of course, is not trivial. It is met in case there is at least one fly in my soup and not met otherwise. What (15b) says, on the other hand, is trivial: since U is the set of everything, it is a superset of the set of flies in my soup, whether or not the latter is empty (given that $\emptyset$ is a subset of every set). It is this semantic difference between (14a) and (14b) which Barwise and Cooper (1981) take to be responsible for the grammatical contrast between these sentences.

The two cases we have just examined, (7) and (14), illutrate the claim that the language system interacts with logic. More specifically, it corroborates Logicality, which states that trivialites are filtered out as ill-formed. The cases pertain to the difference in distribution between universal and existential quantifiers and show how sentences can be analyzed so that a grammatical contrast aligns with a logical contrast: well-formed sentences are contingent, ill-formed sentences are trivial. Similar explanations have been proposed for many other phenomena, among which are the distinction between mass and count nouns (Chierchia, 1998, 2010), the distinction between individual-level and stage-level predicates (Magri, 2009), the distribution of polarity items (Krifka, 1995; Chierchia, 2013; Crnič, 2019), free choice (Menéndez-Benito, 2005; Crnič and Haida, 2020), numerals (Bylinina & Nouwen, 2018; Haida & Trinh, 2020, 2021), comparatives (Gajewski, 2008a), island effects (Fox & Hackl, 2006; Abrusán, 2007), question embedding (Uegaki & Sudo, 2017), just to name a few. The body of works carried out under the perspective of Logicality is large and growing.

I will now turn to the discussion of a problem for Logicality and a solution to it. This solution will be the key to resolving the inconsistency in Wittgenstein’s position on trivialities.

The problem might have become quite obvious to the astute reader, who will have noticed something strange in what we have said so far. On the one hand, we claim that sentences such as (7b) and (14b), repeated below as (16a) and (17a), are ill-formed because they have the trivial entailments in (16b) and (17b), respectively. 
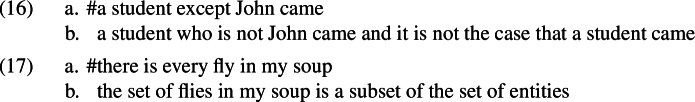


On the other hand, we seem to have no problem accepting these trivial entailments themselves, or more precisely, the sentences we use to convey them, as perfectly grammatical. Neither (16b) nor (17b) are ill-formed. But how do we square this fact with Logicality? Doesn’t Logicality predict all four sentences in (16) and (17) to be ungrammatical? The problem, in fact, can be illustrated more simply, but more dramatically, by the sentences in (6), repeated below. 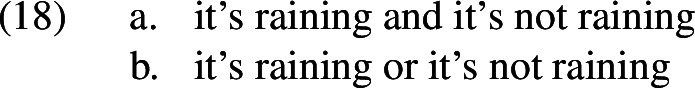


If Logicality is correct, grammar should mark both of these sentences as ill-formed, given that (18a) is a contradiction and (18b) a tautology. But it clearly doesn’t. We can debate the reasons why someone would use (18a) or (18b), but we cannot deny that that person speaks English. Our perception of someone who produces (16a) or (17a), on the other hand, is very different: we would think that he has not mastered English. Thus, (18a) and (18b) are clearly well-formed in a way that (16a) and (17a) are not.

The problem for Logicality, then, is that it excludes more sentences than it should. It predicts all trivialities to be ungrammatical, while in fact only some are. To solve this problem, we have to find a way to distinguish between trivialities that are well-formed, such as (18a) and (18b), and trivialities that are ill-formed, such as (16a) and (17a). The solution I am going to present is one proposed by Del Pinal (2019); Pistoia-Reda and Sauerland (2021); Del Pinal (2022), among others. It is sometimes refered to as “contextualism”.^29^ What it says is that natural language grammar contains a covert, context-sensitive “rescaling” operator, R_c_, which attaches to non-logical expressions and modulates their meaning. The logical form of (18a) is then not (19a) but (19b). 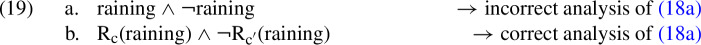


Similarly, the logical form of (18b) is not (20a) but (20b). 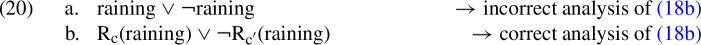


Note, importantly, that one and the same expression may attach to differently indexed rescaling operators. In other words, we allow non-logical terms to shift their meaning within the same sentence. The meaning of R_c_(raining), then, does not have to be identical to that of R_c'_(raining). This means (18a) has a non-trivial reading as saying ‘it is raining in some sense and not raining in some other sense’. In the same way, (18b) has a non-trivial reading. These readings kick in and rescue the sentences from being trivial, hence from being ill-formed. Speakers, however, may not be consciously aware of this process, just as they are not conciously aware of the fact that (16a) and (17a) are ill-formed because the former entails the contradiction in (16b) and the latter the tautology in (17b). The interaction between universal grammar and the natural deductive system takes place at a subconscious level. It results in intuitions on acceptability which speakers can viscerally experience but cannot explicate.

The question now is why the same rescaling process does not kick in and rescue (16a) and (17a) from being trivial, making them well-formed. Well, the answer is that even if it does kick in, it still cannot rescue these sentences from being trivial. Recall a condition on the rescaling operator: it can only attach to non-logical terms. Thus, it would not attach to *except*, *every*, *a*, and *there*, which are logical constants. Let us look at the logical form of (16a) and (17a). 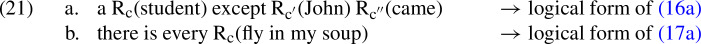


The reader is invited to verify for herself that both (21a) and (21b) are trivial. Given the semantics proposed in (9), *a*(P except E)(Q) is contradictory under all values of P, E, and Q. Similarly, the semantics proposed in (15) predicts that *there*(every(P)) is tautologous under all values of P. Rescaling does not help in these cases.

I will end this section by mentioning an issue which the proponents of Logicality must address and which is also raised by Wittgenstein in the Tractatus. This is the issue of how to distinguish between logical and non-logical constants? For Wittgenstein, the logical constants are those expressions that will disappear in the proper semantic analysis: what they express, namely entailment relations, would emerge from the pictorial nature of the symbols in the ideal notation. Until we succeed in constructing such a notation, however, we have to deal with logical constants in our non-ideal language. Wittgenstein says of such expressions that they “do not represent” (4.0312). This description more or less captures our intution about such words as *every*, *a*, *except*, and *there*. The rescaling story works under the assumption that these words are logical constants. It is true, in some sense, that they do not “represent” anything. However, this description turns out to be too vague. It is not clear what would prevent me from saying, for example, that *every* represents the relation ‘is a subset of’, which is the set of pairs $<\text {X}, \text {Y}>$ such that X $\subseteq$ Y. We need a more rigorous definition of logical constants.

It turns out, however, that such a definition is quite difficult to formulate.^30^ Gajewski (2003), based on ideas from previous works, suggests to define logical constants in terms of “permutation invariance”. Logical constants, then, would be those expressions whose denotation remains constant across permutations of individuals in the domain (Mautner, 1946; Mostowski, 1957; Tarski, 1986; van Benthem, 1989; McGee, 1996). This definition, for example, would include *every* in, and exclude *student* from, the set of logical constants. To illustrate, suppose our domain is $\{a, b, c\}$, where *a* and *b* are students while *c* is not. The denotation of *student* is then $\{a, b\}$. This set will become a different set, $\{b, c\}$, under the permutation $[a$ $\rightarrow$ $b, b$ $\rightarrow$ $c, c$ $\rightarrow$ $a]$, which means *student* is not permutation invariant, hence not a logical constant. In contrast, *every* will denote the set in (22). 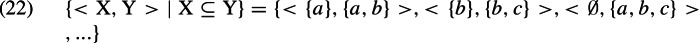


This set will obviously remain the same under all permutations, as switching the individuals around does not affect whether a set is a subset of another set: $\{a\}$ will still be a subset of $\{a, b\}$, hence the pair $<\{a\}, \{a, b\}>$ will still end up in the denotation of *every*, for example. This means *every* is a logical constant, a good result. Gajewski’s suggestion is widely known and cited. However, it clearly cannot be the whole story, as it classifies predicates like *exists* or *is self-identical*, which supposedly denote the universe of discourse U, as logical,^31^ even though these do not incur ill-formedness as we would expect (Abrusán, 2019; Del Pinal, 2022). 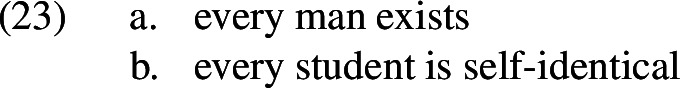


No matter how we modulate the meaning of *man* and *student*, the sets they denote will be a subset of U. There would then be no reading of these sentences in which they are not trivial. However, neither (23a) nor (23b) is ill-formed. This means that *exists* and *self-identical* are in fact not logical constants, as far as the language system is concerned, and that (23a) and (23b) can be analyzed as (24a) and (24b), where R_c_(exists) and R_c_(self-identical) do not denote U. 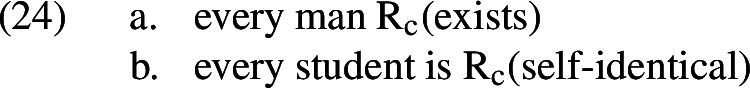


To the best of my knowledge, an adequate intensional definition of logical constants is still missing. At this stage, a wealth of data find illuminating explanations in terms of Logicality. These explanations appeal to an intuitive notion of “logical constants”, but the distinction they draw between logical and non-logical terms is, in the end, stipulative. It is, of course, also possible that the distinction will turn out to be essentially stipulative (Quine, 1951). The jury is still out.

## Transformative analysis

Let us recap. In section 1, I argue that Wittgenstein takes trivialities to be well-formed even though the theory of language which he proposes, PTL, predicts them to be ill-formed. My hypothesis is that Wittgenstein’s inconsistency is rooted in his inadequate analysis of a class of natural language expressions: he analyzes a sentence such as (25), which is perceived to be perfectly well-formed, as having the logical form in (25a), which is a triviality. In section 2, I present a thesis recently developed in theoretical linguistics, Logicality, which provides a way to overcome this inadequacy: Logicality makes it possible, in fact necessary, to analyze (25) not as (25a) but as (25b), which is not a triviality. 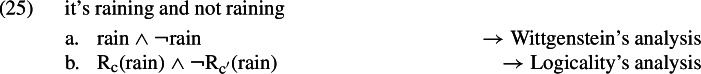


In this section, I will comment on some points of contact between analytic philosophy and theoretical linguistics which have been brought into relief by our discussion.

Analytic philosophy began with the insight that the “logical form” of a sentence, i.e. one which captures its semantic properties, might be quite different from its “surface form”, i.e. one which captures its syntactic, morphological and phonological properties. To illustrate, consider the sentence in (26). Its surface form is (26a), while its logical form would be (26b).^32^
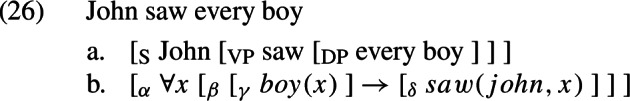


One can see how (26a) and (26b) differ with respect to their constituency. For example, there is no constituent of (26b) which corresponds to the object DP *every boy* in (26a). There are, in addition, four sentential constituents in (26b) – i.e. $\alpha$, $\beta$, $\gamma$, and $\delta$ – while there is only one such constituent in (26a), namely S.^33^ The idea that (26b) could in principle be how (26) looks at some level of description constitutes a “revolution” in the way we think about natural language sentences: semantic analysis must be carried out on a structure different from one which inputs pronunciation or writing. Beaney (2016, p. 235) puts it succinctly: “[T]here is no decomposition without interpretation”. The term “interpretation” here is to be understood as ‘translation’ or ‘transformation’. Thus, semantic analysis consists of (i) the step of “transformative analysis” which translate the surface form of the sentence into its logical form, and (ii) the step of “decompositional analysis” which dissects the logical form and computes its consequences (Beaney, 2000, 2002, 2003, 2016, 2017).^34^

It is at the step of transformative analysis, I submit, that Wittgenstein commits an error with sentences such as those in (18). He takes it for granted that the two instances of *rain* are one and the same symbol. It never occured to him to see this as a case of homophony, i.e. a case of two different symbols, R_c_(*rain*) and R_c'_(*rain*), having one and the same pronunciation. Curiously, Wittgenstein does discuss homophony in the Tractatus: “In everyday language it occurs extremely often that the same word signifies in different ways – that is, belongs to different symbols” (3.323). He even provides an example: “In the proposition ‘Green is green’ – where the first word is a person’s name, the last an adjective – these words [...] involve different symbols” (3.323). Note that the homophonous symbols in this case, “Green” and “green”, belong to different syntactic categories (noun and adjective). It is also obvious that the meaning of the first, the person named Green, and the meaning of the second, the color green, are different. Thus, it is much easier to see homophony here than to see it in the case of R_c_(*rain*) and R_c'_(*rain*), where the two symbols not only belong to the same syntactic category (verb), but also have meanings which differ in a much more subtle way (‘rain in one sense’ and ‘rain in another sense’).

Beaney’s dictum, that there is no decomposition without interpretation, implies that a sentence is associated with at least one structure which inputs pronunciation and one other structure which inputs interpretation. Transformative analysis is the step that relates the two. As it happens, the idea that a sentence is associated with more than one structure is foundational to modern linguistics. It brought about the “generative revolution” in the 1950’s (cf. Chomsky, 1988). In the current “minimalist” version of generative grammar which has established itself more or less as cannonical (Chomsky, 1991, 1995; Radford, 2004), a sentence is associated with two structures: a “phonological form” (PF) and a “logical form” (LF). PF inputs pronunciation while LF inputs interpretation. PF and LF are related by “transformational rules” which build complex structures from lexical items step by step.^35^ Note that the LF of a sentence, just like the logical form which results from transformative analysis, can differ drastically from how we hear the sentence or see it written on paper. For example, the LF of (26) is (27) (Heim & Kratzer, 1998; Fox, 2000, 2003). 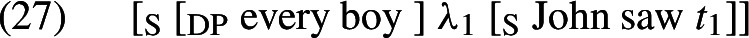


We can thus witness an interesting parallel between the “analytic revolution” in philosophy and the “generative revolution” in linguistics. A notable fact is that the former came much earlier. Its beginning can be dated to Frege’s 1879 debut, *Begriffsschrift*, wherein he proposes quantificational predicate logic (Beaney, 2016, 228). The beginning of generative grammar, in contrast, came with Chomsky’s (1955) magnum opus *The Logical Structure of Linguistic Theory*, wherein he proposes transformations. And it would take the linguists about 20 years more to come up with the idea of LF as a structure which disambiguates scopal relations between quantificational elements in the sentence (May, 1977; Chomsky, 1981; Huang, 1982; May, 1985).^36^ What is the reason for this delay?

The answer, I believe, lies in the difference between early analytic philosophers and generative grammarians with respect to their view on the surface form of natural language sentences. For the former, it is more or less a mess. Serious investigation can only begin after transformative analysis, which is carried out by the philosopher contemplating on what the sentence means. However, the step of transformative analysis is itself shrouded in mystery: there is no theory of it. Coming up with the logical form of a sentence is, therefore, similar to scientific discovery in its spontaneous and revelatory nature. In contrast, generative grammarians take the relation between PF and LF to be systematic: both are derived by transformational rules, which are codified in a unified theory. This theory imposes constraints on possible sound–meaning associations and can thus account for intuitions which, I think, would remain puzzling to the philosophers. Take the phenomenon of “weak crossover”, for example. It is observed that (28a) can be associated with the meaning expressed by (28b) but (29a) cannot be associated with the meaning expressed by (29b). It is not clear how analytic philosophers would explain this contrast. Generative grammarians, on the other hand, have proposed several accounts of it (Koopman & Sportiche, 1983; Reinhart, 1983; May, 1985). 
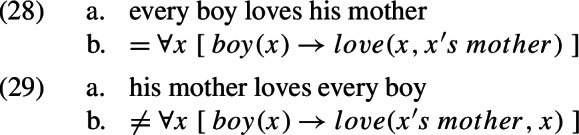


Part of generative grammar, then, can be seen as a proposal on how transformative analysis really works, thereby providing a missing link in analytic philosophy, which considers this step a black box. This would also make sense of the fact that the grammarians’ LF came long after the philosophers’ logical form: it is understandable that it takes more time to figure out *how* something works than to realize *that* it works.

Is there any missing link in generative grammar which is provided by analytic philosophy? At this point, I can only say “maybe”. Recall what Logicality claims: trivialities are ill-formed. The unacceptability of sentences excluded by Logicality is, crucially, of a different kind from that of sentences which violate “pragmatic” rules. To illustrate, compare (30a) and (30b). 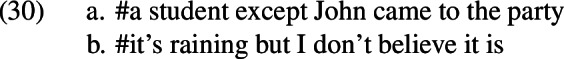


Both sentences in (30) sound “odd”. However, there is a clear difference. A person who produces (30b) might be making a joke, or trying to be philosophical, or having some sort of mental problem, or all three, but she is speaking English.^37^ Her sentence is well-formed. In contrast, a person who produces (30a) is not speaking English. Whatever rules out (30a), therefore, must be a component of formal grammar. Obviously, it is not phonology or morphology. There are no illegitimate combinations of phonemes or morphemes in (30a). This means (30a) fails at syntax. There are two possibilities for a sentence to “fail at syntax”. The first is that it cannot be derived by syntactic rules: formal properties of the words just do not allow them to be put together to build the sentence. An apt metaphor for this scenario would be a computer constructed with parts that do not fit. The second possibility is that the sentence can be derived by syntactic rules but the output “crashes” at the interface between syntax and the interpretive systems: it is not accepted by these systems as legitimate input. A metaphor for this scenario would be a computer which is built with parts that fit perfectly but which has no power port. In both cases, we have a defective product of syntax. It is syntactic defect that gives sentences such as (30a) their distinctive feel of “gibberish”. As far as I know, proponents of Logicality share the view that sentences such as (30a) fail in the second way: they can be derived by the syntactic rules but crash at the syntax-semantics interface. In other words, semantics imposes a “contingency constraint” on the output of syntax (Abrusán, 2019). I have not encountered claims in the literature to the effect that trivialities cannot be derived by syntactic rules, and it is not hard to see why. Syntactic rules are commonly assumed to be “blind” to interpretation. What they care about is whether something is a determiner, not whether its quantificational force is universal or existential. In that sense, syntactic rules do not distinguish between *a* and *every*.

A question that has not been raised, to the best of my knowledge, is *why* there should be a contingency constraint on the output of syntax. Why should structures which express trivialities be markedss as defective syntactic products? Analytic philosophy, I surmise, might help us towards settling this explanatory question. Specifically, the picture theory of language might provide a starting point for developing an answer to it. Thus, suppose we say that interpretation of syntactic structures means mapping them into expressions of a “translation language” TL which is, as it were, the “language of thought”.^38^ And suppose, furthermore, that TL is “pictorial” in the sense envisioned by Wittgenstein. Then trivialities would be marked as defective products of syntax for the reason that they are “untranslatable”. This is, of course, speculative. My objective in this paper is to point out a tension in Wittgenstein’s theory of language and argue that it can be resolved in light of recent advances in linguistic research. This objective, I believe, can be achieved independently of whether my speculation about TL has merits. I do hope, however, that future work will show that it does.^39^

## A loose end

I have proposed that Wittgenstein’s exclusion of trivialities from nonsense is due, in part, to his intuition about the well-formedness of such sentences as (31). 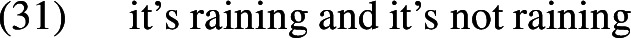


However, Wittgenstein considers such sentences as (32) nonsensical (4.1272).^40^
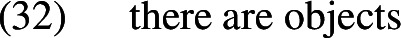


The existence of objects is a precondition for the meaningfulness of language (5.552). This precondition is “shown” by our use of the language. Attempts at “saying” it in the language would result in nonsense. Saying anything means picturing it, which implies it is contingent. But (32) is not contingent: we cannot picture it being false, simply because picturing is not possible if it is false.^41^

But (32) is intuitively well-formed, just like (31). The question that arises, then, is whether my proposal is contradicted by the fact that Wittgenstein takes (32), but not (31), to be nonsensical? The answer, I submit, is no. I never claim intuitive well-formedness guarantees exclusion from nonsense. I only hypothesize that it helps. This hypothesis is compatible with (32) being deemed nonsensical by Wittgenstein. In fact, I would argue that Wittgenstein’s differential treatment of (31) and (32) supports the general theme of my proposal, which is that natural language intuition gives rise to issues in PTL that can be resolved by Logicality. The issue here is that both (31) and (32) are non-pictorial, but Wittgenstein only regards (32) as nonsense. Can we locate the source of this issue in natural language intuition? And can Logicality resolve it?

The answer to both questions, I believe, is yes. Consider (31). This sentence is derived from *it’s raining*, which is clearly pictorial, by (successive) application of negation and conjunction. This means that if Wittgenstein is to declare (31) nonsensical, he would have to “overcome” not only the natural language intuition that it is well-formed, but also the natural language intuition that (33) is true. 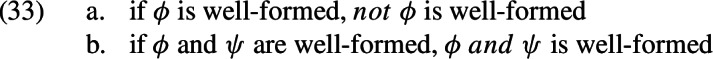


If we accept that *it’s raining* is well-formed, and accept that (33) is true, then we have to accept that (31) is well-formed. And it is overwhelmingly intuitive that (33) is true. The words *not* and *and*, intuitively, are sentential operators: they do not map sentences to non-sentences. Now consider (32). This expression, according to Wittgenstein, fails at the word level. It has no subconstituent which is pictorial. The word *object* is already meaningless, in the sense that it has no translation in the ideal notation. This means that declaring (32) nonsensical does not require abandoning (33). The only natural language intuition which must be overcome is that it is well-formed.

Thus, we can, arguably, locate the source of Wittgenstein’s differential treatment of (31) and (32) in natural language intuition, namely his intuition about *not* and *and*. Now let us ask whether the technology of Logicality, i.e. rescaling, would “resolve the issue”, i.e. would make it possible for Wittgenstein to treat (31) and (32) in the same way. The answer, clearly, is yes. Given rescaling, Wittgenstein could say, for both (31) and (32), that they are contingent under the rescaled analysis and trivial under the non-rescaled analysis.^42^ And the fact that there is one analysis under which they are contingent is responsible for our experience of them as well-formed.

As far as I know, non-pictorial sentences which Wittgenstein deems nonsensical are all of the same type as (32): classifying them as ill-formed does not require abandoning (33). To the extent that I am correct, the above considerations apply generally.

## Conclusion

Wittgenstein’s picture theory of language (PTL) unites grammar and logic as two different symptoms of the same underlying condition. A consequence of PTL, which Wittgenstein refuses to accept, is that trivialities are ill-formed. This turns out to be precisely what is claimed by Logicality, a recently developed thesis about natural language which has been corroborated by a large amount of empirical arguments. Logicality provides an explanation for Wittgenstein’s dissonant attitude towards trivialities. The discussion on PTL and Logicality brings out reasons to take a closer look at the historical relationship between analytic philosophy and generative linguistics as well as the way in which these two disciplines can complement each other going forward.

## Footnotes

1 Or the Tractatus for short. This is the Latin rendition of the original German title (Logisch-philosophische Abhandlung), and was suggested by Moore as title for the English translation of Wittgenstein (1921). There are two well-known printed English translations. The first, published in 1922, was mostly the work of Frank P. Ramsey, even though Charles K. Ogden appeared as the translator. The second, by David F. Pears and Brian F. McGuiness, was published in 1961, with a revised version appearing in 1974. The publisher, in both cases, was Routledge and Kegan Paul (see Michael Beaney’s Introduction chapter in Wittgenstein 2023). There is a new English translation by Michael Beaney, Wittgenstein (2023), which is published by Oxford University Press. In what follows I will use Beaney’s translation, unless otherwise indicated. See note 4.

2 This is what Noam Chomsky means by “generative grammar” (cf. Chomsky, 1955, 1957, 1965, 1986).

3 Wittgenstein originally intended the title of his book to be simply “Der Satz” (“The Sentence”) (cf. Goldstein 2002, citing Bartley III 1973.) Note that although Wittgenstein clearly distinguishes between legitimate and illegitimate combination of symbols, i.e. between sentences and non-sentences, he does not consistently use the term “grammar” in the way I am using it, but alludes to this distinction in a variety of ways. The term he most often uses to refer to non-sentences is “Unsinn” (nonsense). Combinations of symbols that are not nonsense are sentences. We will come back to this term below.

4 The German word “Satz” used by Wittgenstein in the Tractatus is rendered as “proposition” in the English translations. I will, however, use “sentence” as the English counterpart of “Satz”, as I think it conveys more faithfully what Wittgenstein means with “Satz”, namely the syntactic material in its semantic interpretation, not just the semantic interpretation: “[D]er Satz ist das Satzzeichen in seiner projektiven Beziehung zur Welt” (3.12). It seems to me that the word “proposition” has come, especially in the linguistic literature, to denote what the sentence expresses in abstraction from its syntax: two different sentences may express one and the same proposition. The reader will thus be asked to keep in mind that “proposition” in the quotes from Beaney’s English translation, which is the one I use here, and “sentence” in my own text, are just two different renderings of “Satz” in the German original.

5 The discussion is illustrative and the simplistic example I use will undoubtedly beg a lot of questions. I will not attempt to address these questions by adding a footnote qualifying virtually every clause in the next paragraphs. My hope is that the reader can muster enough charity to see the point I want to make without feeling too frustrated by the extreme informality and lack of precision in my exposition.

6 I adhere to the view that Wittgenstein takes “objects” to include properties and relations (Stenius, 1960; Hope, 1965), as long as these are considered basic and thus to be represented by monolithic symbols. For a different view see Copi (1958), Anscombe (1959).

7 In what follows I will refer to sentences in (3) by using the name of the relevant language and the numbers in the first columns. Thus, L_F_-2 is $\lnot s(n)$, L_W_-3 is the jar with the pebble inside and the marble outside of it, for example.

8 Wittgenstein also proposes the idea of illustrating PTL using arrangements of physical objects: “The essence of a propositional sign becomes very clear if we think of it as composed of spatial objects (such as tables, chairs, books) instead of written signs. The mutual spatial position of these things then expresses the sense of the proposition” (3.1431). Thus, Tractarian objects are not formless: they come with constraints on how they combine with other objects (Hope, 1969; Hintikka, 1994, 2000).

9 The syntax is usually set up in such a way that it contains rules for constructing sentences and a “restriction clause” which states that “nothing else is a sentence”, i.e. that only those structures which can be generated by the rules which have been provided are sentences. This clause excludes non-sentences.

10 We can, of course, “imagine” a (tiny) jar being inside a (huge) marble. But I am illustrating PTL with real objects, not imaginary objects. There is no point in using imaginary objects to illustrate the difference between L_F_ and L_W_, as combining imaginary objects would be, of course, as unconstrained as combining letters. I believe, also, that the “tables, chairs, books” that Wittgenstein is talking about in 3.1431 are real, not imaginary (see note 8): he is not talking about imaginary tables and chairs that could be inside of imaginary books. I thank an anonymous reviewer to drawing my attention to the need to clarify this point.

11 The rule in (4a) is sometimes called “simplication”. The logic, just like the syntax, is usually set up in such a way that it contains rules of inference and a restriction clause stating that “no other inference is valid” (see note 9).

12 The reader might wonder whether L_W_-1 entails Mary doesn’t smoke, as it shows that only the pebble is inside the jar, i.e. that only John smokes. This is certainly an intuition that we have about L_W_-1. It is here that our simple example breaks down, the confound being how we, as “cooperative” language users, tend to enrich the message conveyed by a sentence. Specifically, we tend to take the sentence to say not only that the states of affairs it represents are facts but also that these facts are all the facts (Grice, 1967). Thus, the sentence *John smokes*, as an answer to *who smokes?*, is most naturally interpreted as saying not only that John smokes but also that no one else smokes (Groenendijk & Stokhof, 1984; Heim, 1994; Chierchia et al., 2012; Trinh, 2019). In the case of L_W_-1, we tend to see it as conveying not only that the pebble is inside the jar, but also, as conveying that the marble is not inside the jar. Note that the issue then arises as to why we seem not to see L_W_-1 as saying that the marble is not outside the jar. Given that we do not see the marble in L_W_-1 at all, supplying the picture mentally with the marble being inside the jar and supplying it mentally with the marble being outside the jar should be two equally good options. The linguistic counterpart of this issue is known as the “symmetry problem” (Kroch, 1972; Fox, 2007; Fox & Katzir, 2011; Trinh & Haida, 2015; Trinh, 2018, 2019). To the best of my knowledge, no studies have been done on how a “pictorial” variant of this issue, as informally presented here, might help adjudicate between competing proposals, especially regarding the notion of “conceptual alternatives” (Breheny et al., 2018; Buccola et al., 2022). This whole topic deserves a lot more discussion than I am able to provide in this short note. I hope to return to it on another occasion.

13 We could revise the syntax of L_F_ as to disallow the conjunction of a sentence and its negation. However, such a revision would be completely ad hoc.

14 Another way to write $\lnot (s(j) \wedge \lnot s(j))$ is $s(j) \vee \lnot s(j)$, given the definition of $\vee$ as the dual of $\wedge$.

15 As we know, the practice of filling a gap in a chain of reasoning by a conjecture is common in mathematics.

16 See Chomsky (1981). Basically, the $\Theta$-Criterion requires that the number of syntactic arguments of a predicate match exactly the number of its logical arguments. The Socrates example is presented in 5.473 and 5.4733.

17 I thank an anonymous reviewer for drawing my attention to this line of thinking.

18 I thank an anonymous reviewer for pointing this out.

19 The same argument can be made with respect to the term “Elementarsatz” (atomic sentence): when Wittgenstein says “Satz”, he means ‘Satz’, not ‘Elementarsatz’. In fact, this is the main argument of Eisenthal (2023).

20 I should stress, also, that an inconsistency does not necessarily make a theory “bad” or mean that its proponents are intellectually inferior. The Standard Model is inconsistent with General Relativity, but physical theory remains the crown jewel of scientific achievement by the greatest minds. An inconsistency may arise from several theses, each of which seems unavoidable, but all of which cannot be simultaneously true, as is the case, I believe, with A, B and C in Wittgenstein’s theory of language. This can make the theory fruitful, as it stimulates further thought and investigation. I provide this footnote in awareness of possible reactions to my paper as shown by one colleague who, after I presented it, said to me: “Wittgenstein is not an idiot!” Of course Wittgenstein is not an idiot. He is a deep thinker whose ideas foreshadowed one of the most important discoveries in linguistics. His contribution would have been greatly diminished, I think, if he retreats into the safe space of consistency and restricts his picture theory to sentences which obviously pose no challenge to this theory.

21 Again, I note that “well-formed” and “ill-formed” are not terms used in the Tractatus. I hope, however, that my use of these terms, given the explanation I just gave, will cause no confusion. I thank an anonymous reviewer for pointing out the need to make it clear that “ill-formed” is not used by Wittgenstein.

22 As pointed out, interestingly, by Peters and Westerståhl (2023), the observation about exceptives was made as early as in the 14th century by William Ockham: “An exceptive proposition is never properly formed unless its non-exceptive counterpart is a universal proposition. Hence, *a man except Socrates is running* is not properly formed” (*Summa Logicae* Part II:18, translated by Alfred J. Freddoso and Henry Schuurman).

23 The reader will notice that I am mixing object and metalanguage. My use of *every*, for example, is ambiguous between the expression and the relation it denotes. I hope no confusion results from this informality.

24 We assume that *student* denotes the set of students, i.e. the set $\{x$ | *x* is a student$\}$, and the name *John* can be “type-shifted” to denote the singleton set containing John, i.e. the set $\{$John$\}$ (Partee, 1986).

25 The reader can also verify that (9) explains not only the contrast between *every* and *a* but that between universal quantifiers (e.g. *every*, *all*, *no*) and existential quantifiers (e.g. *a*, *some*, *most*) in general. Note, also, that my presentation of von Fintel (1993) differs from the original in one minor detail: whereas I take the phrase *except John* to be a modifier of *student*, von Fintel takes it to be a modifier of the determiner. This difference has no bearing on the point being made. Since von Fintel (1993) other accounts of exceptive constructions have been proposed, most of which also explain the ill-formedness of (7b) by reducing it to a contradiction (Gajewski, 2008b; 2013; Crnič, 2021; Vostrikova, 2021), but some of which explain it in other ways (Moltmann, 1995; Hirsch, 2016).

26 This example is taken from Abrusán (2019). To the best of my knowledge, the general observation goes back to Milsark (1977).

27 We will assume, for simplicity, that the copula *is* is not interpreted (Heim & Kratzer, 1998).

28 Assuming that *fly in my soup* denotes the set of flies in my soup, i.e. $\{x$ | *x* is a fly in my soup$\}$.

29 Gajewski (2003) proposes another solution which is in the same spirit but differ in technical implementation. For arguments that contextualism is a better solution see Sauerland (2017); Pistoia-Reda and Sauerland (2021); Del Pinal (2022).

30 For an overview of attempts at characterizing the notion of a “logical constant” see MacFarlane (2017).

31 Obviously, U remains the same under all permutations of the individuals in it.

32 The representation in (26b) is Frege’s analysis, written in modern notation (Frege, 1879; Beaney, 2016).

33 Another example of this approach is Frege’s analysis of numerical statements (Frege, 1884; Reck, 2007; Levine, 2007). The most well-known example is perhaps Russell’s (1905) analyis of definite descriptions: the surface form of (i) is (ia) while its logical form is (ib).  According to Russell’s analysis, (i) says that there is exactly one king of France who is bald. The analysis predicts the sentence to be meaningful, i.e. truth-valuable, without France being a monarchy. This is an empirical prediction, and we can debate in what way it is correct and in what way it is not. For analyses of definite descriptions which differ from Russell’s in that they do not make this prediction, see Frege (1884, 1892); Strawson (1950); Heim (1982, 1991).

34 Other terms Beaney uses for transformative analysis are “explicatory analysis” and “interpretive analysis” (Beaney, 2007, 2016).

35 These transformations inlude *merge*, the single syntactic rule which operates on semantically interpreted units, and rules of morphology and phonology which operate on units of pronunciation (Hauser et al., 2002; Chomsky, 2007, 2008, 2013).

36 In the beginning of generative grammar, semantic interpretation was read off of the so-called “deep structure” (Chomsky, 1957, 1965), which is different from what is latter called LF.

37 The sentence in (30b) exemplifies what is called “Moore’s Paradox”, as it was G. E. Moore who noticed that sentences of the form $\phi$ *but I don’t believe that* $\phi$ are odd (Moore, 1942). It can be shown that given Grice’s 1967 maxims of conversations and standard assumptions about belief, the speaker of (30b) has contradictory beliefs (Schlenker, 2016).

38 For such a translation approach to semantic interpretation see Montague (1973).

39 The greatest challenge to a theory of TL would seem to be intensional contexts: *A thinks* *p*, *must* *p*, etc. Wittgenstein suggests to analyze *A thinks* *p* as depicting a situation where two configurations of objects mirror each other, one of which represents, presumbaly, A’s thought and the other represents *p* (5.542). Thus, *A thinks* *p* describes a fact about the world, just like *it is raining*. To the extent that such an extensional analysis is conceivable, there is no principled reason for insisting that a pictorial representation is impossible in this case. The same argument can be made for modals. In fact, the “double relative” analysis of modality, which has more or less become consensus among linguists, would say that a sentence like *John must pay a fine* depicts, for example, a situation where John has parked his car in the driveway and the relevant rules specify that parking one’s car in the driveway incurs a fine (Kratzer, 1977, 1981, 1991; von Fintel & Heim, 2011). I thank an anonymous reviewer for raising the issue of modal sentences.

40 I thank an anonymous reviewer for pointing out that sentences such as (32) should be discussed.

41 To see how (32) cannot be said in a Tractarian pictorial language, imagine we have to use the language discussed in section 1 – with the pebble (John), the marble (Mary), and the jar (smoking) – to say that John exists. Obviously, we cannot. Note, importantly, that holding up the pebble, which is the symbol for John, is not “saying” anything. The natural language counterpart to that would be uttering just the word *John*. Of course, such an utterance may be understood as saying something, if the word *John* is interpreted as if it is part of a sentence. Suppose the question *who smokes?* has been asked, for example. Uttering *John* in response would then amount to saying that John smokes. This example, clearly, does not show that words can be meaningful in isolation. In fact, it shows the opposite: it corroborates Frege’s “context principle” (Frege, 1884), which Wittgenstein also assumes, that a word can only be interpreted as part of a sentence (2.0122). The fact that sentences in natural language can be elliptical, i.e. can contain parts that are not pronounced, presents no challenge to this principle.

42 The word *object* can be rescaled to mean ‘physical objects in the visual field’, for example.
